# FORTIFYING SCHOLARSHIP IN THE PUBLIC INTEREST: AN EXAMINATION OF THE MARGINALIZATION OF DRAG ARTISTS

**DOI:** 10.1093/geroni/igae098.0746

**Published:** 2024-12-31

**Authors:** Lyn Holley, Kenneth Hites

**Affiliations:** University of Nebraska at Omaha, Omaha, Nebraska, United States; University of Nebraska Omaha, Omaha, Nebraska, United States

## Abstract

Over 14 states have passed or are considering legislation that severely restricts and criminalizes public drag performances. Some bills erroneously label the artistry as “adult cabaret performances” thus, conflating them with nude performances. The Americans Civil Liberties Union, Movement Advancement Project, and other organizations have declared a state of emergency due to this endangerment of constitutionally protected individual human rights. 7.2 to 8 % of the U.S. adult population identifies as sexual and gender minorities (SGM) (HRC, 2021, Gallup,2023). Drag artists are a smaller population within SGM communities and it is believed that the language of anti-drag legislation is designed to also oppress transgender individuals (a separate SGM community). Overall, 2024 has seen 478 anti-LGBTQ bills, 21 of which are drag-specific Several prominent scholars (e.g., Steven Miller of Clemson) have called attention to the similarity between these attacks and the process of “othering” used by Hitler to create public acceptance of annihilation of Jews, the disabled, sexual and gender minorities and ultimately, members of any politically or economically inconvenient group (e.g., older, retired adults). This individual presentation provides a framework to guide scholarly observation and understanding of the current attacks and clarifies the relevant contributions that only scholars can make to produce scientifically valid information and knowledge about Drag Artists and their social and economic impacts on USA society and the economy, thereby strengthening and informing decisions about laws and policy that govern individual freedom of expression.

